# Familial Longevity Is Not Associated with Major Differences in the Hypothalamic–Pituitary–Gonadal Axis in Healthy Middle-Aged Men

**DOI:** 10.3389/fendo.2016.00143

**Published:** 2016-11-09

**Authors:** Evie van der Spoel, Ferdinand Roelfsema, Steffy W. Jansen, Abimbola A. Akintola, Bart E. Ballieux, Christa M. Cobbaert, Gerard J. Blauw, P. Eline Slagboom, Rudi G. J. Westendorp, Hanno Pijl, Diana van Heemst

**Affiliations:** ^1^Section Gerontology and Geriatrics, Department of Internal Medicine, Leiden University Medical Center, Leiden, Netherlands; ^2^Section Endocrinology, Department of Internal Medicine, Leiden University Medical Center, Leiden, Netherlands; ^3^Department of Clinical Chemistry and Laboratory Medicine, Leiden University Medical Center, Leiden, Netherlands; ^4^Section Molecular Epidemiology, Department of Medical Statistics, Leiden University Medical Center, Leiden, Netherlands; ^5^Department of Public Health, Center of Healthy Aging, University of Copenhagen, Copenhagen, Denmark

**Keywords:** luteinizing hormone, testosterone, hypothalamic–pituitary–gonadal axis, familial longevity, hormone secretion, approximate entropy, temporal correlation

## Abstract

**Context:**

A trade-off between fertility and longevity possibly exists. The association of the male hypothalamic–pituitary–gonadal (HPG) axis with familial longevity has not yet been investigated.

**Objective:**

To study 24-h hormone concentration profiles of the HPG axis in men enriched for familial longevity and controls.

**Design:**

We frequently sampled blood over 24 h in 10 healthy middle-aged male offspring of nonagenarian participants from the Leiden Longevity Study together with 10 male age-matched controls. Individual 24-h luteinizing hormone (LH) and testosterone concentration profiles were analyzed by deconvolution analyses to estimate secretion parameters. Furthermore, the temporal relationship between LH and testosterone was assessed by cross-correlation analysis. We used (cross-)approximate entropy to quantify the strength of feedback and/or feedforward control of LH and testosterone secretion.

**Results:**

Mean [95% confidence interval (CI)] total LH secretion of the offspring was 212 (156–268) U/L/24 h, which did not differ significantly (*p* = 0.51) from the total LH secretion of controls [186 (130–242) U/L/24 h]. Likewise, mean (95% CI) total testosterone secretion of the offspring [806 (671–941) nmol/L/24 h] and controls [811 (676–947) nmol/L/24 h] were similar (*p* = 0.95). Other parameters of LH and testosterone secretion were also not significantly different between offspring and controls. The temporal relationship between LH and testosterone and the strength of feedforward/feedback regulation within the HPG axis were similar between offspring of long-lived families and controls.

**Conclusion:**

This relatively small study suggests that in healthy male middle-aged participants, familial longevity is not associated with major differences in the HPG axis. Selection on both fertility and health may in part explain the results.

## Introduction

Over the last decades, several conserved mechanisms have been identified that associate with longevity in both animal models and humans. The common function of these evolutionarily conserved systems is to enable the organism to adequately respond to changes in the environment in order to maintain homeostasis. This is achieved by adapting the balance between growth, development, and reproduction versus maintenance and repair (1). In mammals, these adaptive responses are centrally regulated and involve the hypothalamus. The hypothalamus regulates homeostasis *via* neural and endocrine pathways. These latter comprise the hypothalamic–pituitary–thyroid (HPT) axis, the hypothalamic–pituitary–growth hormone (GH) axis, the hypothalamic–pituitary–adrenal (HPA) axis, the hypothalamic–pituitary–prolactin (PRL) axis, and the hypothalamic–pituitary–gonadal (HPG) axis. To maintain homeostasis, these centrally regulated hormonal axes respond to stressors, such as inflammation and starvation. As a response to systemic inflammatory disease, the HPA axis is activated and available resources are reallocated by suppressing the GH, HPG, and HPT axes among others (2). Likewise, fasting was observed to suppress the HPG, HPT, GH, and PRL axes and to stimulate the HPA axis in healthy men (3). While these adaptive responses are clearly beneficial for short-term survival, their long-term health consequences may vary depending on the type and severity of the stress. Extension of health and lifespan can also be induced by genetic mutations altering one or more neuroendocrine axes (4, 5). In model organisms, many long-lived mutants have a reduced reproductive output. Also in humans, decreased reproduction was found to associate with exceptional human longevity in both men and women (6).

It is well known that the lifespan of men is shorter than that of women. It has been speculated that enhanced exposure to male sex hormones and/or decreased exposure to female sex hormones may be contributing factors. Estrogens in women were found to upregulate the expression of antioxidant, longevity-related genes, which could be a biological explanation for sex differences in longevity (7). In line, a later age of menopause was associated with reduced female mortality (8). In men, the HPG axis involves hypothalamic gonadotropin-releasing hormone (GnRH), luteinizing hormone (LH) from gonadotrophs in the anterior lobe of the pituitary gland and testosterone from the Leydig cells of the testis. Little is known about direct effects of the male HPG axis on human longevity. Studies in castrated men are inconclusive; Korean eunuchs had a longer lifespan than non-castrated men of similar socioeconomic status, whereas the lifespan of castrated singers was similar to that of non-castrated singers (9, 10). Longevity inducing interventions, such as fasting, lead to a decline in LH secretory burst mass and testosterone concentrations and to an increase in LH release pattern orderliness (3, 11). However, the association of reduced testosterone secretion or other characteristics of the male HPG axis with human familial longevity has not been investigated yet.

In order to identify determinants of human longevity, the Leiden Longevity Study (LLS) included offspring of long-lived families that are enriched for exceptional longevity and partners thereof, serving as a control group (12). To date, the 24-h profiles of three hypothalamic hormonal axes have been investigated in detail in the LLS. It was found that TSH secretion was higher and TSH–fT3 temporal relationship was stronger in the offspring compared to controls (13, 14). Furthermore, we found that GH secretion was diminished and tightly controlled in offspring compared to controls (15). However, we did not find an association between familial longevity and cortisol secretion under resting conditions (16). In the current study, we test whether male sex hormones are associated with familial longevity by comparing 24-h LH and testosterone secretion parameters, the temporal relationship between LH and testosterone, and the strength of feedforward and feedback control signals within the HPG axis between male offspring of long-lived families and age-matched controls.

## Materials and Methods

### Study Population

The LLS comprises 421 families with at least two long-lived Caucasian siblings fulfilling the age criteria (men ≥89 years and women ≥91 years) without selection on health or demographics, as described previously in more detail (12). In the Switchbox Leiden Study, we included 20 offspring of nonagenarian LLS participants together with 18 partners of the offspring as environmental- and age-matched controls (13). Exclusion criteria were having chronic renal, hepatic or endocrine disease, or using medication known to influence lipolysis, thyroid function, glucose metabolism, GH or IGF-1 secretion, and/or any other hormonal axis. Moreover, participants were excluded based on the presence of fasting plasma glucose >7 mmol/L, recent transmeridian flight, smoking addiction, or extreme diet therapies. To be able to safely perform the 24-h blood sampling, other exclusion criteria were difficulties to insert and maintain an intravenous catheter, anemia (hemoglobin <7.1 mmol/L), and blood donation within the last 2 months. Based on the information obtained *via* telephone questioning, controls with a nonagenarian parent who had one or more nonagenarian siblings were also excluded. Participants were middle-aged (52–76 years) and had a stable body mass index (BMI) between 18 and 34 kg/m^2^. The Switchbox Leiden Study was approved by the Medical Ethical Committee of the Leiden University Medical Centre and was performed according to the Helsinki declaration. All participants gave written informed consent for participation.

### Clinical Protocol

Full details on the 24-h blood sampling procedure have been described previously (17). In short, a catheter was placed in a vein of the forearm of the non-dominant hand and, starting around 09:00 hours, 3.2 mL of blood was collected every 10 min. Participants received standardized feeding consisting of 600 kcal Nutridrink (Nutricia Advanced Medical Nutrition Zoetermeer, The Netherlands) at three fixed times during the day. Participants were not allowed to sleep during the day, and except for lavatory use, no physical activity was allowed during the study period. Lights were switched off for approximately 9 h (circa between 23:00 and 08:00 hours) to allow the participants to sleep. Height and weight were measured in the research center. BMI was calculated as weight (in kilograms) divided by the square of height (in meters). Body composition was determined by bioelectrical impedance analysis (BIA) at a fixed frequency of 50 kHz (Bodystat^®^ 1500 Ltd., Isle of Man, British Isles) (18). Waist circumference was measured with a measuring tape midway between the uppermost border of the iliac crest and the lower border of the costal margin.

### Hormone Assays

All hormonal assays were performed with fully automated equipment and diagnostics from Roche Diagnostics (Almere, The Netherlands) at the Department of Clinical Chemistry and Laboratory Medicine of the Leiden University Medical Centre in The Netherlands, which is accredited according to the National Coordination Committee for Quality Assurance for Health Care Laboratories in The Netherlands. LH (catalog number 11732234122) and testosterone (catalog number 05200067190) were measured in EDTA plasma samples collected every 10 min using ECLIA (Electrochemoluminiscentie immunoassay) on a Roche Modular E170 immunoanalyser. The measuring range of LH is 0.100–200 U/L, and the interassay coefficients of variation (CV) were 4.47% at 4.10 U/L and 2.83% at 56.43 U/L. The measuring range of testosterone is 0.087–52.0 nmol/L, and the interassay CV were 4.12% at 1.77 nmol/L and 3.78% at 34.84 nmol/L. Testosterone is mostly bound to binding proteins including sex hormone-binding globulin (SHBG) and albumin. Albumin (catalog number 11970909216) and SHBG (catalog number 03052001190) were measured on Roche Modular analyzers in six EDTA plasma samples with 4-h intervals for each participant. Free testosterone was calculated based on albumin and SHBG levels as described by Vermeulen et al. (19).

### Additional Blood Measurements

Approximately 2 weeks before the study day, fasting serum was withdrawn to screen for factors that might have an impact on testosterone production. Dehydroepiandrosterone sulfate (DHEAS) with catalog number L2KDS2 was measured using a solid-phase competitive chemiluminescent enzyme immunoassay with an Immulite 2000 XPi system from Siemens Healthcare diagnostics (The Hague, The Netherlands). Total 25-hydroxyvitamin D (catalog number 05894913190) was measured using ECLIA with an E170 module of Modular Analytics from Roche Diagnostics. Interleukin 6 (catalog number SS600B) and tumor necrosis factor-α (TNF-α) (catalog number SSTA00D) were measured by ELISA from R&D Systems. High-sensitivity C reactive protein (hsCRP) with catalog number 04628918190 was determined by a particle-enhanced turbidimetric assay using Cobas Integra 800 from Roche Diagnostics.

### 24-h Pool Measurements

For each participant, 3 μL of all (144) samples taken during the 24-h blood sampling were pooled. In this mixture, the levels of testosterone (catalog number 05200067190), estradiol (catalog number 06656021190), and prolactin (catalog number 03203093190) were determined using ECLIA and E170 module of Modular Analytics from Roche Diagnostics.

### Deconvolution Analysis

To determine underlying components of LH and testosterone secretion, 24-h LH and testosterone concentration profiles were analyzed by validated deconvolution analysis (20). By deconvolution analysis, a hormone concentration profile is decomposed into underlying secretory bursts, basal secretion, elimination of previously secreted hormone, and random experimental variability. The algorithm in the Matlab software program (Mathworks, Inc., Natick, MA, USA) first detrends the data and normalizes concentrations to numbers within the interval 0–1. Thereafter, successive potential pulse-time sets, each containing one fewer burst, were created by a smoothing process. Finally, a maximum-likelihood expectation deconvolution method estimated all secretion and elimination rates simultaneously for each candidate pulse-time set. Outcome parameters of main interest are basal (non-pulsatile) secretion, pulsatile secretion, the sum of basal and pulsatile secretion (total secretion), number of pulses per 24 h (secretory burst frequency), interpulse regularity (Weibull gamma), mean pulse mass, and slow half-life. For LH, fast half-life was fixed to 6.93 min and slow half-life was estimated as unknown variable between 40 and 120 min (21). For testosterone, fast and slow half-lives were fixed to 1.4 and 27 min, respectively (22).

### Approximate Entropy

Jack-knifed approximate entropy (JkApEn) is a measure for the strength of feedforward and feedback control signals in a hormone system. First, approximate entropy (ApEn), which is a scale- and model-independent statistic that quantifies the regularity of consecutive time-series data, was calculated using the Matlab software program (Mathworks, Inc., Natick, MA, USA) (23). ApEn has high sensitivity and specificity (both >90%) for analysis of hormone concentration measurements over 24 h. Low ApEn values imply that the sequence of time-series data is regular and that it contains many repetitive patterns. High ApEn values indicate greater irregularity and randomness. Normalized ApEn parameters of *m* (window length) = 1 and *r* (criterion of similarity) = 20% of the SD of the individual subject time series were used (24). Subsequently, jackknifing was performed, which is a rigorous and objective leave-one-out cross validation test that gives less bias in smaller samples than regular ApEn, and it is more applicable for hormone data (25).

### Cross-Approximate Entropy

Bivariate cross-ApEn is a scale- and model-independent regularity statistic, which quantifies the relative pattern synchrony of two time series (26). Changes in the cross-ApEn reflect feedback and/or feedforward alterations within an interlinked axis, with the cross-ApEn of LH-T representing feedforward asynchrony and cross-ApEn of T-LH indicating feedback asynchrony (27).

### LH/T and T/LH Ratios

Besides ApEn, other proxies for the strength of the feedforward and feedback signaling in the HPG axis are the ratios of LH and testosterone concentrations. LH/testosterone ratio reflects the feedback of testosterone on LH secretion, with a higher ratio signifying lower feedback. Testosterone/LH ratio reflects the strength of the feedforward drive of LH on the testosterone secretion, with a higher ratio denoting higher feedforward (28).

### Cross-Correlation

The temporal relationship between LH and testosterone concentrations was determined by cross-correlation. Cross-correlation assesses the relative strength between a hormone pair for all possible time shifts. For the offspring and control groups, the strongest correlation coefficient with corresponding lag time was compared.

### Statistical Analysis

Descriptive statistics was used to summarize the characteristics of study groups. The non-parametric Mann–Whitney *U*-test and independent-samples *T*-test were used to assess differences between offspring and controls in, respectively, variables that were not normally distributed and normally distributed variables. LH and testosterone secretion parameters were compared between offspring and controls using linear regression adjusted for age. Variables were examined for normality. Variables that were not normally distributed were logarithmically transformed prior to analysis and are presented as geometric means with 95% confidence intervals (CIs), except for two variables, LH slow half-life and testosterone total secretion, because logarithmic transformation did not improve normality. *p* ≤ 0.05 was considered statistically significant. All statistical analyses were performed using the SPSS for Windows, version 23 (SPSS, Chicago, IL, USA). Graphs were made using the GraphPad Prism version 6 (GraphPad, San Diego, CA, USA).

## Results

### Group Characteristics

Group characteristics of male offspring of long-lived families and controls are presented in Table 1. Participants were selected based on the age of one of their parents. Consequently, the parents of the offspring were significantly older on average (*p* = 0.02) than those of the controls. Offspring and controls were similar in age, body composition, available immunological markers, DHEAS, and 25-hydroxyvitamin D. Group characteristics of female offspring of long-lived families and controls are presented in Table S1 in Supplementary Material. Similar to men, female offspring and controls did not differ significantly in age, body composition, available immunological markers, DHEAS, and 25-hydroxyvitamin D, except for mean age of the parents.

**Table 1:** **Group characteristics of male offspring of long-lived families and controls**.

	Offspring *n* = 10	Controls *n* = 10	*p*-Value
Age (years)^a^	66.6 (6.4)	64.6 (4.0)	0.41
BMI (kg/m^2^)^a^^,^^b^	26.0 (3.4)	25.7 (3.2)	0.84
Height (cm)^b^	177 (175–182)	181 (175–184)	0.60
Fat mass (kg)^b^	19.8 (16.4–25.2)	18.5 (18.1–23.6)	0.84
Lean body mass (kg)^b^	61.8 (56.5–66.2)	60.5 (58.3–66.3)	0.72
Waist circumference (cm)^b^	96.5 (88.3–109.8)	97.0 (93.0–104.0)	0.91
Mean age of parents (years)	89.0 (83.4–95.0)	79.5 (74.6–84.3)	**0.02**
DHEAS (μmol/L)	4.0 (1.5–5.9)	2.6 (2.0–6.4)	0.74
Vitamin D (nmol/L)	71.9 (53.0–103.0)	69.9 (56.5–85.6)	0.91
Interleukin 6 (pg/mL)	0.8 (0.6–1.3)	1.1 (0.9–1.6)	0.22
TNF-α (pg/mL)	1.7 (1.4–4.0)	1.4 (1.3–2.2)	0.22
hsCRP (mg/L)^c^	0.7 (0.6–3.0)	0.9 (0.6–1.2)	0.83

*Unless indicated otherwise, data are presented as median with interquartile ranges*.

*Bold values indicate p ≤ 0.05*.

*^a^Data are presented as mean with SD*.

*^b^Data were not available for one control subject due to technical problems*.

*^c^Two controls were excluded with hsCRP >20 (indicative of acute inflammation) approximately 2 weeks before the study day*.

### LH and Testosterone Secretion

To illustrate the wide variation between participants in LH and testosterone concentration profiles, Figures S1 and S2 in Supplementary Material present the individual 24-h LH and testosterone concentration profiles of all participants, respectively. Results of the deconvolution analyses on 24-h concentration profiles of plasma LH and testosterone are shown in Table 2. Mean (95% CI) total LH secretion of the offspring was 212 (156–268) U/L/24 h and that of controls 186 (130–242) U/L/24 h, which did not differ significantly (*p* = 0.51). Likewise, basal LH secretion, pulsatile LH secretion, and characteristics of pulsatile LH secretion were similar between the groups. Mean (95% CI) total testosterone secretion of the offspring was 806 (671–941) nmol/L/24 h, which was similar (*p* = 0.95) to the mean (95% CI) total testosterone secretion of controls [811 (676–947) nmol/L/24 h]. Other parameters of testosterone secretion were also not significantly different between the offspring and controls.

**Table 2:** **LH and testosterone secretion parameters in male offspring of long-lived families and controls**.

	Offspring *n* = 10	Controls *n* = 10	*p*-Value
**LH**			
Mean LH (U/L)	5.1 (3.9–6.4)	5.3 (4.0–6.5)	0.89
Slow half-life (min)^b^	59.7 (46.6–72.8)	69.9 (56.7–83.0)	0.27
Total secretion (U/L/24 h)	212 (156–268)	186 (130–242)	0.51
Basal secretion (U/L/24 h)	134 (94–175)	116 (76–156)	0.51
Pulsatile secretion (U/L/24 h)^a^	69.9 (51.8–94.3)	64.5 (47.8–87.0)	0.70
Number of pulses (per 24 h)	14.4 (13.1–15.7)	13.7 (12.4–15.0)	0.46
Interpulse regularity γ^a^	2.2 (1.9–2.6)	2.2 (1.9–2.7)	0.88
Mean pulse mass (U/L)	5.3 (4.0–6.5)	5.1 (3.9–6.4)	0.85
**Testosterone**			
Mean testosterone (nmol/L)^a^	14.2 (12.1–16.8)	13.8 (11.7–16.2)	0.78
Mean calculated free testosterone (nmol/L)^a^	0.43 (0.38–0.49)	0.40 (0.35–0.45)	0.39
Total secretion (nmol/L/24 h)^b^	806 (671–941)	811 (676–947)	0.95
Basal secretion (nmol/L/24 h)	608 (502–714)	619 (513–726)	0.88
Pulsatile secretion (nmol/L/24 h)	198 (138–258)	192 (132–252)	0.88
Number of pulses (per 24 h)	23.0 (19.3–26.6)	21.7 (18.1–25.4)	0.62
Interpulse regularity γ	2.6 (2.1–3.1)	2.3 (1.9–2.8)	0.44
Mean pulse mass (nmol/L)	8.3 (6.3–10.2)	8.8 (6.8–10.8)	0.69

*Unless indicated otherwise, data are presented as mean with 95% confidence interval and analyzed by linear regression adjusted for age*.

*^a^Data are presented as geometric mean with 95% confidence interval*.

*^b^Results of the Mann–Whitney U-tests were similar to results of linear regression*.

### Feedforward and Feedback Regulation of LH and Testosterone Secretion

Approximate entropy of LH and of testosterone, representing the strength of feedback and feedforward regulation, were similar between offspring and controls, as shown in Table 3. LH-T cross-ApEn, reflecting feedforward synchrony, was similar between offspring of long-lived families and controls. Equally, T-LH cross-ApEn, reflecting feedback synchrony, was not significantly different between the groups. Furthermore, mean LH/T ratio, representing feedforward drive, and mean T/LH ratio, representing feedback, were similar between offspring and controls.

**Table 3:** **Proxies of feedforward and feedback regulation of LH and testosterone (T) secretion in male offspring of long-lived families and controls**.

	Offspring *n* = 10	Controls *n* = 10	*p*-Value
LH ApEn	1.5 (1.3–1.7)	1.4 (1.2–1.6)	0.54
T ApEn	1.8 (1.6–1.9)	1.6 (1.4–1.7)	0.11
LH-T cross-ApEn (feedforward asynchrony)	1.8 (1.6–1.9)	1.6 (1.4–1.8)	0.40
T-LH cross-ApEn (feedback asynchrony)	2.0 (1.8–2.2)	1.8 (1.6–2.0)	0.25
Mean LH/T ratio^a^ (feedback)	0.3 (0.3–0.5)	0.4 (0.3–0.5)	0.75
Mean T/LH ratio (feedforward)	3.6 (2.6–4.6)	3.1 (2.2–4.1)	0.50

*Unless indicated otherwise, data are presented as mean with 95% confidence interval*.

*^a^Data are presented as geometric mean with 95% confidence interval. Analyses are adjusted for age*.

### Temporal Relationship of LH and Testosterone

The maximal correlation between LH and testosterone concentrations was found at a lag time between 60 and 80 min with a correlation coefficient (*r*) of 0.16 for offspring and *r* = 0.29 for controls, which was not significant different (*p* = 0.21) when adjusted for age.

### Measurements in 24-h Pool

Because all women were postmenopausal, determination of sex hormones was limited to a single measurement in the 24-h pool. No differences were observed in testosterone, estradiol, and prolactin levels between offspring and controls, neither in men nor in women (Table 4).

**Table 4:** **Testosterone, estradiol, and prolactin in a 24-h pool for offspring of long-lived families and controls**.

	Men			Women		
	Offspring *n* = 10	Controls *n* = 10	*p*-Value	Offspring *n* = 10	Controls *n* = 8	*p*-Value
24-h testosterone (nmol/L)	15.4 (12.1–18.9)	14.3 (12.2–18.0)	0.85	0.47 (0.35–1.14)	0.31 (0.16–0.49)	0.24
24-h estradiol (pmol/L)	81.7 (60.5–97.2)	83.2 (66.2–96.4)	0.99	15.3 (9.2–37.7)	9.2 (9.2–27.7)	0.57
24-h prolactin (μg/L)	8.9 (7.8–9.5)	8.4 (7.4–9.7)	0.63	10.4 (8.5–12.8)	10.7 (9.6–13.8)	0.76

*Data are presented as median with interquartile ranges and analyzed with Mann–Whitney U-tests*.

## Discussion

In this study, we did not observe significant differences in 24-h secretion of HPG axis parameters and their regulation between male subjects enriched for familial longevity compared to controls. LH and testosterone secretion parameters, and the strength of feedforward and feedback regulation within the HPG axis, were similar between offspring of long-lived families and controls. Also, testosterone, estradiol, and prolactin levels measured in a 24-h pool were similar between the offspring and controls in men and in women.

According to the disposable soma theory, which suggests a trade-off between fertility and longevity, longevity would be associated with reduced reproductive capacity, i.e., reduced HPG axis action (1). However, we observed in the current study that male offspring of long-lived families did not differ from age-matched controls in their HPG axis. There are several potential explanations for the observed absence of an association between male HPG axis and familial longevity.

Possibly, this observation is related to the selection criteria of the study population. First, due to the recruitment of the study population based on the presence of at least two long-lived siblings, we may have selected on fertility, which is strongly associated with the HPG axis. Second, we applied strict exclusion criteria based on health. Health is highly associated with the HPG axis; health status can influence the HPG axis, and conversely, the HPG axis can influence health. Inflammation is a major factor influencing the HPG axis, which was also shown recently by Veldhuis et al. reporting on healthy male subjects receiving an IL-2 injection which led to an immediate decrease in testosterone secretion (28). Even though it is widely accepted that starting at the age of around 30–40 years, testosterone levels decline relatively linearly with age, some studies have shown that testosterone does not significantly decline with age in exceptionally healthy men (29–31). It is unclear whether age-related pathophysiological features, which are associated with a decline in testosterone, are a consequence of testosterone decline or partly the cause. However, testosterone supplementation can reverse some of these pathophysiological features, indicating that testosterone itself has a major influence on age-related pathophysiology (32). Because of the strict exclusion criteria on health, the two groups of men were very similar in their health status, as also indicated by similar markers of health, and possibly consequently also in their HPG axis.

Supporting our findings, no mutations in the HPG axis have been associated with changes in lifespan in model organisms, indicating that a constitutively lower activity of the HPG axis is likely not associated with longevity. Long-living IGF-1 and GH-mutant mice do show suppression in HPG hormones and reproduction, but mainly in female mice (33). However, suppression of HPG hormones and reproduction might not be essential for their longevity phenotype, but rather be a pleiotropic effect of reduced GH/IGF-1 signaling. Moreover, the possible trade-off between fertility and longevity could be only present in women and not in men. This could be explained by the fact that the amount of energy invested in reproduction is much higher in women than in men. However, the possible trade-off in women seems not to be linear, but U-shaped, with an optimum of having approximately two children (34). In the current study, we did not find an association between sex hormones and familial longevity in women. However, we were limited to a single measurement in postmenopausal women. Thus, testing this hypothesis should preferably be performed in women with a younger age. Finally, the HPG axis might be too important for fitness, thus preventing the spread of mutations that would constitutively downregulate this axis. It has even been argued that because men are able to reproduce up to high ages, late-life male reproductive success would drive a positive association between fertility and longevity (35).

One of the limitations of the current study was the relatively small sample size. This study is underpowered to detect many small differences but adequately sampled to identify major differences in hormonal axes. Indeed, in the same study population, comprising men and women, offspring had a 59.9% higher total TSH secretion and a higher temporal correlation between TSH and fT3 compared to controls (13, 14). Furthermore, GH secretion was lower and tighter controlled in the offspring compared to controls (15). When restricted to men only, which is a sample identical to the sample of men used in the current study, differences of similar magnitude were found in TSH secretion, temporal TSH–fT3 correlation, GH secretion, and GH ApEn.

Inherent to the study design of the LLS, in which families are included based on two proband nonagenarian siblings, the age range of the offspring was 52–76 years. Therefore, LH and testosterone profiles could not be obtained at young age. Another limitation of this study was that there could have been dilution of potential differences between offspring and controls, since possibly not all offspring may have inherited the favorable predisposition for longevity of their long-lived parent. Strength of our study is that we sampled blood every 10 min during 24 h, creating the opportunity to study the HPG axis in detail. Moreover, we performed our study in a special cohort, in which we were able to study human familial longevity.

To conclude, this relatively small study suggests that in healthy male middle-aged participants, human familial longevity is not associated with major differences in the HPG axis.

## Author Contributions

Conceptualization and methodology: DH, FR, RW, and HP; formal analysis: ES, FR, SJ, and DH; investigation: SJ, AA, and ES; resources: BB and CC; writing – original draft: ES and DH; writing – review and editing: ES, SJ, AA, BB, CC, PS, GB, RW, HP, FR, and DH; visualization: ES and DH; supervision: DH, HP, and FR; funding acquisition: RW, DH, and HP.

## Conflict of Interest Statement

The authors declare that the research was conducted in the absence of any commercial or financial relationships that could be construed as a potential conflict of interest.
