# Supplementary material for: Familial Longevity Is Not Associated with Major Differences in the Hypothalamic–Pituitary–Gonadal Axis in Healthy Middle-Aged Men
Source: Front Endocrinol (Lausanne). 2016 Nov 9;7:143. doi: 10.3389/fendo.2016.00143 (PMC5101217; doi:10.3389/fendo.2016.00143)
Supplement: Supplementary file 1 [file Table_1.PDF]

Table S1. Group characteristics of female offspring of long-lived families and controls

	Offspring n=10	Controls n=8	p-value
Age (years)‡	64.7 (4.4)	64.5 (6.1)	0.95
BMI (kg/m^2)	23.5 (21.6 – 30.6)	23.1 (21.1 – 28.9)	0.99
Height (cm)	164 (161 – 168)	167 (161 – 171)	0.57
Fat mass (kg)	23.5 (19.7 – 34.7)	23.5 (19.1 – 34.6)	0.97
Lean body mass (kg)	42.0 (36.8 – 44.7)	40.4 (37.7 – 47.4)	0.90
Waist circumference (cm)	82.0 (80.3 – 100.3)	86.5 (80.0 – 94.8)	0.83
Mean age of parents (years)	90.3 (82.3 – 93.1)	80.5 (72.3 – 84.0)	**0.02**
DHEAS (µmol/L)*	3.0 (1.8 – 4.7)	1.8 (1.2 – 2.4)	0.07
Vitamin D (nmol/L)*	74.0 (61.4 – 115.3)	85.1 (60.9 – 101.5)	0.96
Interleukin 6 (pg/ml)*	1.1 (0.8 – 2.1)	1.2 (1.0 – 1.8)	0.74
TNF-α (pg/ml)*	1.6 (1.4 – 6.1)	2.0 (1.4 – 4.4)	0.89
hsCRP (mg/L)*	1.1 (0.6 – 3.6)	1.2 (0.7 – 2.0)	0.99

Unless indicated otherwise, data are presented as median with interquartile ranges. ‡Data are presented as mean with standard deviation. *Data were not available for one offspring.
