# Supplementary material for: Familial Longevity Is Not Associated with Major Differences in the Hypothalamic–Pituitary–Gonadal Axis in Healthy Middle-Aged Men
Source: Front Endocrinol (Lausanne). 2016 Nov 9;7:143. doi: 10.3389/fendo.2016.00143 (PMC5101217; doi:10.3389/fendo.2016.00143)
Supplement: Supplementary file 3 [file Image_2.PDF]

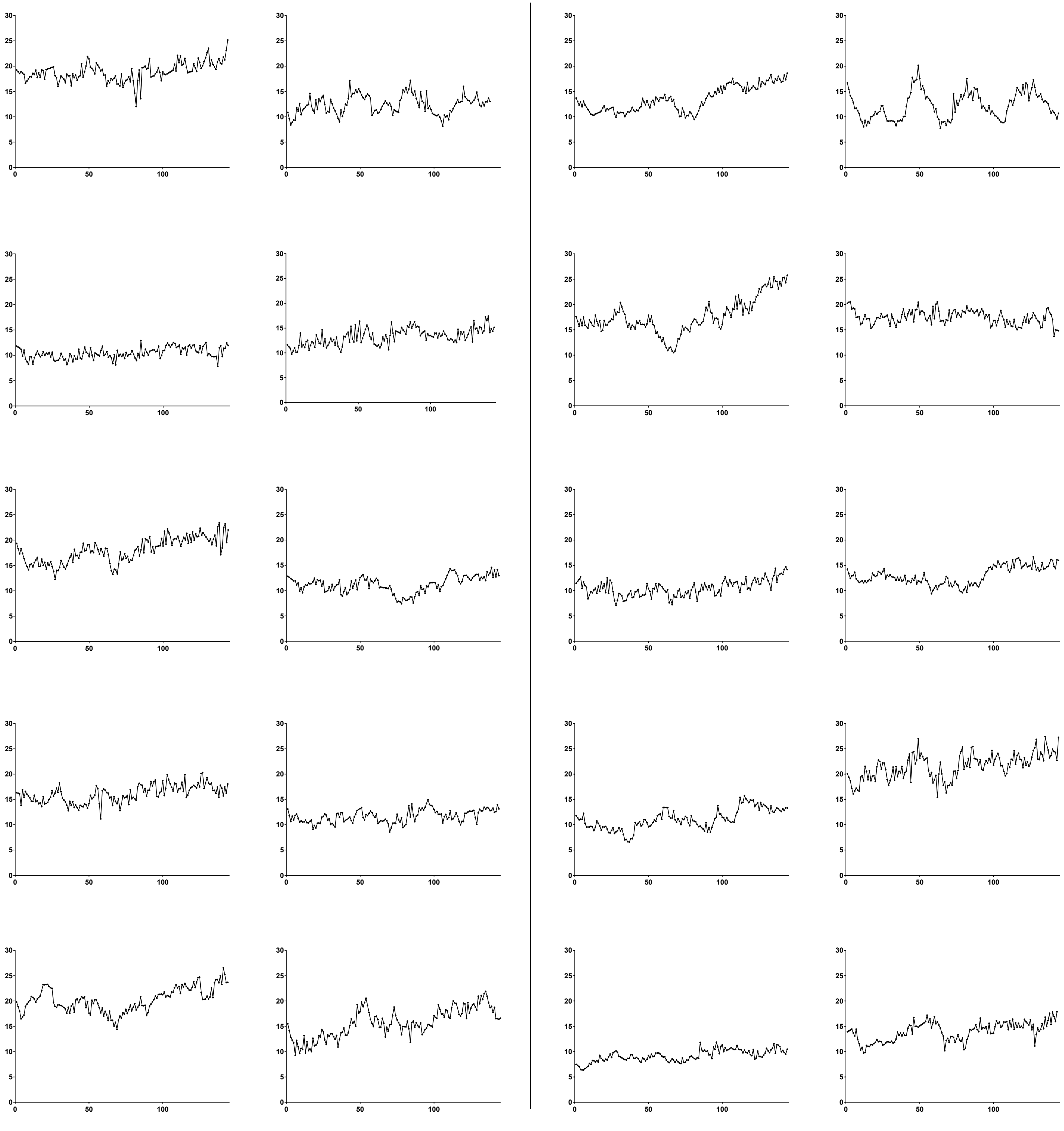

Figure 2. 24-hour testosterone concentration profiles of all participants
Left columns present testosterone concentration profiles of 10 offspring of long-lived families and right columns of 10 controls. Testosterone measurements were performed every 10 minutes for 24 hours, starting around 9:00h. The x-axis presents samplenumber 1 to 144 and the y-axis testosterone concentration in nmol/L.
